# Anthropometric Characteristics in Taiwanese Adults: Age and Gender Differences

**DOI:** 10.3390/ijerph18147712

**Published:** 2021-07-20

**Authors:** Shih-Chang Chen, Chaou-Wen Lin, Po-Fu Lee, Hui-Ling Chen, Chien-Chang Ho

**Affiliations:** 1 Department of Leisure Industry and Health Promotion, National Ilan University, Ilan County, Yilan City 260, Taiwan; ncc5207@niu.edu.tw (S.-C.C.); f520184fred@yahoo.com.tw (P.-F.L.); 2 Department of Marine Leisure and Tourism, Taipei University of Marine Technology, Taipei City 111, Taiwan; mikelin319@mail.tumt.edu.tw; 3 Center of General Education, Taipei Medical University, Taipei City 110, Taiwan; 4 Graduate Institute of Educational Leadership and Development, Fu Jen Catholic University, New Taipei City 242, Taiwan; 024145@mail.fju.edu.tw; 5 Department of Physical Education, Fu Jen Catholic University, New Taipei City 242, Taiwan; 6 Office of Physical Education, Fu Jen Catholic University, New Taipei City 242, Taiwan; 7 Research and Development Center for Physical Education, Health, and Information Technology, Fu Jen Catholic University, New Taipei City 242, Taiwan

**Keywords:** obesity, BMI, WHR, Taiwan

## Abstract

Population aging is creating critical issues in Taiwan, and adults are being forced to maintain productivity at work; in other words, they need to work longer. Therefore, their fitness and health warrant immediate attention. Although the association between health and anthropometric characteristics has been reported, few profiles on Taiwanese adults can be found. The purpose of this study was to provide a suitable reference on the anthropometric data of Taiwanese adults. We recruited 60,056 anthropometric measurements from a representative database. Significant differences were found in every measurement for each gender and age group. Statistically, our results indicated anthropometric differences in different ages. However, CVs showed that the dispersions are minor. This study presents a sufficient profile on Taiwanese adults from a representative database to practitioners and other potential users.

## 1. Introduction

Taiwan has been reported as a super-aged society with a rapidly decreasing adult population [1]. In Taiwan, population aging is creating socioeconomic problems, and its impacts worldwide warrant immediate attention. For example, many countries have adopted pension systems to maintain sustainability [2,3,4,5]. Moreover, the increasing costs of public health services have also challenged society and its general productivity [6]. Thus, the health condition of adults has become more critical in Taiwan since they are responsible for the productivity of society.

Anthropometric parameters are commonly related to one’s physical fitness [7], dietary status [8], lifestyle [9] and general health condition [10,11]. Although anthropometric parameters may be affected by genetic differences, environmental issues and sociocultural conditions, they can still provide significant information on clinical and epidemiological issues [12]. A recent study reported a longitudinal relationship between anthropometric parameters and stress and its tendency to cause overeating [13]. Over the past years, studies have focused on the associations between anthropometric parameters and potential employee selection [14], psychological issues [13], ergonomic product design [15,16] and touchscreen information system design [17]. Even though there are many more indicators that predict human health, anthropometric parameters, such as the body mass index (BMI) and waist–hip ratio (WHR), provide an easy and inexpensive measurement approach for communities [18].

According to the World Health Organization (WHO), anthropometric data should be collected regularly and provided as a standard reference for preliminary health monitoring [19]. These data are recommended to be shown in 10-year clusters and should be distinguished from gender differences [18,19,20]. However, to the best of our knowledge, anthropometric reference data have seldom been provided for adults in Taiwan, and previous studies have focused on adolescents [21] and the elderly [22].

In addition, these studies used a small sample for analysis. Increasing the quality of these data is essential. Even though these data may make a limited contribution to scientific development, sufficient reference data are worth to be established due to their practical value, especially for the adult population. Therefore, this study aimed to provide gender- and age-specific characteristics of anthropometric parameters in Taiwanese adults by using a secondary database.

## 2. Materials and Methods

### 2.1. Study Design and Participants

Cross-sectional analysis was conducted by using the Taiwanese National Physical Fitness Survey database (NPFSIT). The NPFSIT is operated annually in order to survey the physical fitness level of citizens. The government supervises its procedures, data collection, data management and applications. The design, sampling protocols and data validation of the NPFSIT have been previously introduced [23,24,25], and de-identified data from the NPFSIT have been released for research. The data that this study has used were collected from 62,586 participants (29,685 men, 32,901 women) from October 2014 to March 2015. Convenient sampling was applied at 46 examination stations across 22 cities in Taiwan. The purpose and procedure of the NPFSIT were explained to the participants. All participants provided informed consent. The study design and analysis protocol were supervised by the Institutional Review Board of the Fu Jen Catholic University, Taiwan (FJU-IRB C108006).

### 2.2. Data Collection

Before data collection, some regional training seminars were conducted for the examiners to ensure the protocols and assessments could be correctly presented. All the examiners qualified for the training, as reported previously [26,27]. The study was conducted in three phases. The first was to complete the survey questionnaire. The items included sociodemographic characteristics, lifestyle and perceived health status. The second phase was to check each participant’s resting heart rate and blood pressure for safety purposes. Participants whose systolic blood pressure exceeded 140 mmHg or diastolic blood pressure exceeded 90 mmHg or who reported heart disease, hypertension, chest pain, vertigo or musculoskeletal disorders were excluded. The last phase was an anthropometric variable assessment.

The sociodemographic items in the questionnaire were age, gender, education, monthly income and marital status. The lifestyle questions were related to smoking and betel nut chewing. A 5-point Likert scale measured the perceived health status by asking the participants whether they felt healthy. There were three levels of education (elementary school or lower, junior or senior high school and college or higher), monthly income (under 20,000 New Taiwan Dollar (d), 20,001 to 40,000 NTD, and above 40,001 NTD) and marital status (married, never married and divorced/separated/widowed). The participants were also asked whether they never, formerly or currently used cigarettes and/or betel nuts.

### 2.3. Anthropometric Variable Assessment

Anthropometrics were measured for weight, height, waist circumference (WC) and hip circumference (HC). The weight and height were measured by an automatic weight and height machine. The participants were asked to remove their shoes and heavy clothes and stand in a normal posture during measurement. Each WC and HC measurement was performed twice, and the mean value was calculated. The participants were asked to stand in a normal posture, breathe out and hold their breath for a second, and the WC between the lowest rib and the iliac crest was measured. Similarly, the HC was measured as the distance around the widest part of the buttocks (below the hip plates). The Taiwanese health administration recommends that men maintain their WC below 90 cm and women below 80 cm to avoid obesity [28].

The body mass index (BMI) and waist–hip ratio (WHR) were easily calculated. Based on the BMI, the participants were divided into four different groups: underweight, normal weight, overweight and obese. The cut-off points for four groups were 18.5, 24 and 27 kg/m^2^, according to the Health Promotion Administration in Taiwan [29]. The WHR cut-off points were 0.9 for men and 0.85 for women [30].

### 2.4. Statistical Analysis

AS 9.4 software (SAS Institute, Cary, NC, USA) was used to analyze gender- and age-specific data. The statistical measurements included the mean, standard deviation and percentiles (5th, 25th, 50th, 75th, 85th, 90th and 95th). The age groups were 23–24, 25–34, 35–44, 45–54 and 55–64 years. The coefficient of variation (CV) was calculated (standard deviation (SD)/mean) for each anthropometric measurement to determine the dispersion. Means ± standard deviation (SD) or frequency percentages were presented. Student’s *t*-test and chi-square tests were performed for continuous and categorical variables, respectively. Tukey’s post hoc test was used to compare the differences among the groups. The level of significance was set at *p* < 0.05.

## 3. Results

Table 1 shows the demographics and anthropometrics of the 62,586 participants (29,685 men, 32,901 women). Both men and women were significantly different in each measurement (age, body weight, height, BMI, WC, HC, WHR, education, income level, marital status, self-reported health status, smoking status, betel nut chewing; *p* < 0.001).

Table 2, Table 3, Table 4 and Table 5 show the results of each anthropometric parameter (mean, standard deviation, CV and percentile) for both men and women, distributed by age. Significant differences were found in all mean values between genders (*p* < 0.05), and all mean values were higher in men than in women. In addition, mean values in both men and women were significantly different among ages (*p* < 0.05). In men, the mean WC and WHR in the eldest age group were higher than the younger age groups. In women, except for body weight, all the observed means in the eldest age group were significantly higher than in other groups (*p* < 0.05).

The median values (p50) of the body weight and BMI were slightly lower than the means in both men and women. Moreover, the median values of the WC, HC and WHR were slightly lower than the means in women. In general, the result indicated slightly skewed distributions with a wide dispersion for each measurement. Tukey’s multiple comparisons test showed that in men, differences in the WHR were significant between all age groups; in addition, significant differences were found in the height, BMI and WC between the eldest (55–64 years) and youngest (23–24 years) age groups (*p* < 0.05; Table 2 and Table 3). In contrast, the BMI, WC and WHR in women were significantly different in all age groups. In addition, the means of the body weight, height and HC were significantly different between the eldest and youngest age groups (*p* < 0.05; Table 4 and Table 5).

The difference in the mean body weight between the youngest (23–24 years) and oldest (55–64 years) age groups was 0.22 kg (median 0 kg) in men and 2.33 kg (median 3 kg) in women. Tukey’s multiple comparisons test revealed no significant difference between the youngest and oldest age groups. However, the 23–24-year age group was significantly different from all other age groups in men, and the 23–24-year and 25–34-year age groups were significantly different from all the other age groups in women.

The difference in the mean height between the youngest (23–24 years) and oldest (55–64 years) age groups was 6.04 cm (median 6 cm) in men and 4.85 cm (median 5 cm) in women. Tukey’s multiple comparisons test revealed a significant difference between the three older age groups (35–44, 45–54 and 55–64 years) and the two younger age groups (23–24 and 25–34 years) in both men and women. Moreover, the CVs for height were around 0.03 to 0.04 in both men and women. These results presented even distributions in height in the study population (Table 2 and Table 4).

In men, the difference in the mean BMI was 1.64 kg/m^2^ (median 1.9 kg/m^2^) between the youngest (23–24 years) and oldest (55–64 years) age groups. Tukey’s multiple comparisons test revealed a significant difference between the two younger age groups (23–24 and 25–34 years) and the three older age groups (35–44, 45–54 and 55–64 years). In women, the difference in the mean BMI was 2.35 kg/m^2^ (median 2.59 kg/m^2^) between the youngest and the oldest age groups. There were statistically significant differences in all age groups.

The difference in the mean WC between the youngest (23–24 years) and oldest (55–64 years) age groups was 6.86 cm (median 7.4 cm) in men and 7.61 cm (median 9 cm) in women. In both men and women, the WC increased with age. Tukey’s multiple comparisons test showed a significant difference between the two younger age groups (35–44 and 45–54 years) and the three older age groups (35–44, 45–54 and 55–64 years). However, there was no significant difference between the 35–44-year and the 45–54-year age group in men (Table 3), while there were significant differences between all the age groups in women (Table 5).

The difference in the mean HC between the youngest (23–24 years) and oldest (55–64 years) age groups was 0.04 cm (median 1 cm) in men and 1.82 cm (median 2 cm) in women. The HC increased with age in women. Tukey’s multiple comparisons test showed that the 45–54-year age group was significantly different from all other age groups in men, while there were significant differences between all age groups in women (Table 4 and Table 5).

The difference in the mean WHR between the youngest (23–24 years) and oldest (55–64 years) age groups was 0.07 cm (median 0.08 cm) in men and 0.07 cm (median 0.07 cm) in women. In both men and women, the WHR increased with age. Moreover, there was a significant difference between all age groups in both men and women (Table 4 and Table 5).

Table 6 presents the prevalence of BMI categories according to the Taiwanese cut-off points, showing that in both men and women, the normal BMI category was most prevalent (men 40.69%, women 61.72%). However, the percentages of overweight and obese individuals combined were larger in men (57.56%) than in women (32.17%). Looking at BMI categories by age group, it is clear that in both men and women, the highest percentages of underweight people (men 56.28%, women 15.81%) were found in the youngest age group (23–24 years) and the highest percentages of overweight people (men 38.69%, women 28.76%) were found in the oldest age group (55–64 years). In addition, the highest percentages of obese people were found in the oldest age group in women (16.45%) and in the 45–54-year age group in men (25.04%).

Table 7 shows that men had a higher proportion of a normal WC (71.93%) than women (69.35%) but a lower normal WHR (69.44%) than women (76.90%). Both men and women in the youngest age group (23–24 years) had the highest percentages of a normal WC (men 84.95%, women 83.74%) and WHR (men 89.71%, women 89.01%). The oldest age group (55–64 years) had higher percentages of obese people (WC: men 35.01%, women 46.56%; WHR: men 50.56%, women 39.85%).

## 4. Discussion

The purpose of the present study was to provide reference data of gender- and age-specific distributions in Taiwanese adults’ anthropometric parameters. Results showed significant differences in most anthropometric outcomes (weight, height, BMI, WC, HC and WHR) between genders. More importantly, at the weight level, the prevalence of underweight people was 1.75% for men and 6.11% for women, and the prevalence decreased with age. Specifically, there were significant differences in the height, BMI and WC between the youngest and oldest age groups (*p* < 0.05) in women. Differences in the WHR were significant between all age groups.

In anthropometric outcomes, results indicated that all indexes (weight, height, BMI, WC, HC and WHR) of men and women were significantly different in each age group. In addition, men are higher means than women, consistent with previous findings. In a previous study [31], all body dimensions were manually measured using digital calipers and measuring tapes in 100 adults and 100 older people, and the results were the same as the present study. In addition, the mean values in both men and women were significantly different in every age group. In men, the mean WC and WHR were higher in the oldest than the youngest age group. In women, except those for body weight, all the variable means were higher in the oldest age group. This is because aging affects the body composition and metabolism differently between genders, leading to reduced fat oxidation and accumulation of upper-body fat in men and an increased ratio of upper–lower body fat and bone loss in women [32].

WHR results indicated that obesity rates are higher in older people than in the younger population in both men and women. According to the WHO [30], individuals with a higher WHR may have higher abdominal obesity risk. Abdominal obesity is significantly associated with cardiovascular disease [33], risk of cancer [34,35], all-cause mortality [36] and metabolic syndrome [37]. Practitioners should understand that a high WHR could reveal possible health risks for their clients. In addition, promoting a healthier lifestyle could be essential for this population.

On the other hand, although the CVs showed minor dispersions among groups, and the examiners were trained and qualified in the courses, the absolute reliability of the measurements could not be further tested based on the current cross-sectional data. Future studies should be aware of the test reliabilities. For instance, a repeated test may be conducted for the calculation of individual CVs. Thus, the mean CV can be applied to compare the reliability among the measurements [38]. A small mean CV represents a better consistency within each measurement [39].

The strength of the present study was a representative sample from Taiwanese adults. However, there were some limitations. First, the study adopted a cross-sectional design. Thus, no causal relationship could be guaranteed. Future studies should focus on a longitudinal study design to examine sex- and age-related effects on anthropometric development. Second, this study recruited 23–64-year-old Taiwanese adults and cannot be effectively estimated to other populations, such as different ages, races and cultures. Therefore, future studies should survey data from different population groups [40,41] to build a more comprehensive anthropometric profile. Third, as mentioned earlier, although data Heteroscedasticity seemed acceptable by the CV, a measurement error may exist. Lastly, the NPFSIT should widely collect the background of its participants, include more scientific surveys and allow users to connect the data with other sources (e.g., health insurance, medical history) in order to create a better, more comprehensive platform for researchers.

## 5. Conclusions

The anthropometric status provides a preliminary evaluation of one’s health and warrants a suitable profile for reference. The present study used a representative population of Taiwanese adults for analysis and provided details of the anthropometric distribution. Even though differences among different ages and genders have been previously reported, the results provide sufficient profiles to practitioners in Taiwan for both clinical and theoretical purposes.

## Figures and Tables

**Table 1:** Demographic and anthropometric characterization of the study population.

Variables	Total (*N* = 62,586)	Men (*n* = 29,685)	Women (*n* = 32,901)	*p*-Value
Age (years) (%)				<0.001 *
23–24	7.0	8.0	6.1	
25–34	25.4	28.2	23.0	
35–44	27.4	27.6	27.1	
45–54	21.9	20.9	23.0	
55–64	18.3	15.3	20.9	
Body weight (kg)	64.4 ± 12.2	72.2 ± 10.5	57.4 ± 8.8	<0.001 *
Height (cm)	164.2 ± 8.6	170.7 ± 6.3	158.4 ± 5.8	<0.001 *
BMI (kg/m^2^)	23.8 ± 3.5	24.8 ± 3.3	22.9 ± 3.4	<0.001 *
WC (cm)	80.0 ± 9.8	84.5 ± 8.7	75.9 ± 8.9	<0.001 *
HC (cm)	95.5 ± 6.6	96.9 ± 6.2	94.2 ± 6.6	<0.001 *
WHR	0.84 ± 0.07	0.87 ± 0.06	0.80 ± 0.07	<0.001 *
Education level (%)				<0.001 *
Elementary school or lower	3.6	1.7	5.3	
Junior or senior school	27.9	24.1	31.3	
College or higher	68.5	74.1	63.4	
Income level (%)				<0.001 *
≦20,000 NTD	21.0	15.0	26.4	
20,001–40,000 NTD	41.1	34.6	47.1	
≧40,001 NTD	38.0	50.4	26.5	
Marital status (%)				<0.001 *
Never married	54.2	52.9	55.4	
Married	42.1	44.8	39.6	
Divorced/separated/widowed	3.8	2.3	5.1	
Self-reported health status (%)				<0.001 *
Excellent or good	60.8	62.1	59.5	
Fair	32.6	31.6	33.6	
Bad or poor	6.6	6.3	6.9	
Smoking status (%)				<0.001 *
Never	83.8	70.5	95.7	
Current	10.9	19.6	3.0	
Former	5.4	9.9	1.2	
Chewing betel nut				<0.001 *
Never	95.0	90.6	99.0	
Current	2.1	3.5	0.8	
Former	3.0	5.9	0.3	

BMI, body mass index; NTD, New Taiwan dollar; SD, standard deviation; WC, waist circumference; WHR, waist–hip ratio. Values are expressed as means ± SD. * *p* < 0.05.

**Table 2:** Body weight, height and BMI of men aged 23 to 64 years.

Variables	*n*	Mean	SD	CV	p5	p10	p15	p25	p50	p75	p85	p90	p95
Body weight (kg) *^†^													
23–24	2372	69.77 ^a,e^	11.46	0.16	53.0	56.0	58.0	61.3	69.0	77.0	82.0	86.0	91.0
25–34	8363	72.65 ^b^	10.87	0.15	56.0	59.0	61.0	65.0	72.0	80.0	84.0	87.0	93.0
35–44	8205	74.01 ^c^	10.30	0.14	58.0	61.0	63.0	67.0	74.0	81.0	85.0	88.0	92.0
45–54	6198	72.07 ^d^	9.97	0.14	56.7	60.0	62.0	65.0	72.0	78.4	82.0	85.0	90.0
55–64	4547	69.55 ^a,e^	9.47	0.14	54.0	58.0	60.0	63.0	69.0	76.0	79.3	82.0	86.0
Total	29,685	72.20	10.49	0.15	56.0	59.0	61.8	65.0	71.9	79.0	83.0	86.0	91.0
Height (cm) *^†^													
23–24	2372	172.78 ^a,b^	5.86	0.03	164.0	166.0	167.0	169.0	173.0	177.0	179.0	180.0	182.0
25–34	8363	172.46 ^a,b^	5.93	0.03	163.0	165.0	166.1	169.0	172.0	176.0	178.0	180.0	182.0
35–44	8205	171.52 ^c^	6.01	0.04	162.0	164.0	165.1	168.0	172.0	175.9	178.0	179.0	181.0
45–54	6198	169.29 ^d^	6.08	0.04	159.0	162.0	163.0	166.0	169.0	173.0	175.0	177.0	179.0
55–64	4547	166.74 ^e^	6.00	0.04	157.0	159.5	161.0	163.0	167.0	171.0	173.0	174.0	176.0
Total	29,685	170.69	6.33	0.04	160.0	163.0	164.0	167.0	171.0	175.0	177.0	179.0	181.0
BMI (kg/m^2^) *^†^													
23–24	2372	23.35 ^a^	3.58	0.15	18.34	19.10	19.66	20.76	22.94	25.52	27.13	28.36	30.07
25–34	8363	24.41 ^b^	3.34	0.14	19.29	20.32	21.01	22.06	24.11	26.49	27.99	29.03	30.45
35–44	8205	25.14 ^c,d,e^	3.16	0.13	20.20	21.22	21.94	22.94	24.91	27.15	28.41	29.35	30.69
45–54	6198	25.12 ^c,d,e^	3.08	0.12	20.38	21.38	22.04	23.04	24.98	27.01	28.30	29.06	30.48
55–64	4547	24.99 ^c,d,e^	2.97	0.12	20.43	21.45	22.07	23.05	24.84	26.83	28.04	28.84	30.11
Total	29,685	24.77	3.25	0.13	19.71	20.76	21.47	22.53	24.58	26.81	28.08	29.05	30.45

BMI, body mass index; CV, coefficient of variation; SD, standard deviation; p with the ordinal number, percentiles. ^a,b,c,d,e^ Superscripts on the mean values represent Tukey’s test results. Means with the same letter represent that the mean values of the age groups have no significant difference between/among each other. In contrast, different superscript letters show significant differences (*p* < 0.05). * Significant differences in means were found between men and women (Student’s *t*-test; *p* < 0.05). ^†^ Significant differences in means were found across all age groups (ANOVA; *p* < 0.05).

**Table 3:** WC, HC and WHR of men aged 23 to 64 years.

Variables	*n*	Mean	SD	CV	p5	p10	p15	p25	p50	p75	p85	p90	p95
WC (cm) *^†^													
23–24	2372	79.66 ^a^	9.09	0.11	66.0	69.0	70.0	73.0	79.0	85.0	90.0	92.0	96.5
25–34	8363	82.84 ^b^	8.67	0.10	70.0	72.0	74.0	77.0	82.0	89.0	92.0	94.0	98.0
35–44	8205	85.56 ^c,d^	8.37	0.10	72.0	75.0	77.0	80.0	85.0	91.0	94.0	97.0	100.0
45–54	6198	85.85 ^c,d^	8.18	0.10	73.0	75.0	77.5	80.0	86.0	91.0	94.0	96.0	100.0
55–64	4547	86.52 ^e^	8.32	0.10	73.0	76.0	78.0	81.0	86.4	92.0	95.0	97.0	100.0
Total	29,685	84.53	8.70	0.10	70.0	73.0	75.0	78.0	84.0	90.0	94.0	96.0	99.5
HC (cm) *^†^													
23–24	2372	95.92 ^a,e^	7.13	0.07	85.0	87.0	88.5	91.0	95.0	100.0	103.5	106.0	109.0
25–34	8363	97.37 ^b,c^	6.49	0.07	87.0	89.0	91.0	93.0	97.0	102.0	104.0	106.0	108.0
35–44	8205	97.60 ^b,c^	6.09	0.06	88.0	90.0	91.0	93.5	97.5	102.0	104.0	106.0	108.0
45–54	6198	96.47 ^d^	5.87	0.06	87.0	89.0	90.5	92.5	96.0	100.0	102.0	104.0	106.5
55–64	4547	95.96 ^a,e^	5.78	0.06	87.0	89.0	90.0	92.0	96.0	100.0	102.0	103.0	106.0
Total	29,685	96.92	6.24	0.06	87.0	89.0	90.5	93.0	97.0	101.0	103.0	105.0	108.0
WHR *^†^													
23–24	2372	0.83 ^a^	0.05	0.06	0.75	0.76	0.78	0.79	0.82	0.86	0.89	0.90	0.93
25–34	8363	0.85 ^b^	0.05	0.06	0.77	0.78	0.79	0.81	0.85	0.89	0.90	0.92	0.94
35–44	8205	0.88 ^c^	0.05	0.06	0.79	0.81	0.82	0.84	0.88	0.91	0.93	0.94	0.96
45–54	6198	0.89 ^d^	0.05	0.06	0.80	0.82	0.84	0.86	0.89	0.92	0.94	0.96	0.98
55–64	4547	0.90 ^e^	0.06	0.07	0.81	0.83	0.85	0.87	0.90	0.94	0.96	0.97	0.99
Total	29,685	0.87	0.06	0.07	0.78	0.80	0.81	0.83	0.87	0.91	0.93	0.94	0.97

BMI, body mass index; CV, coefficient of variation; SD, standard deviation; p with the ordinal number, percentiles. ^a,b,c,d,e^ Superscripts on the mean values represent Tukey’s test results. Means with the same letter represent that the mean values of the age groups have no significant difference between/among each other. In contrast, different superscript letters show significant differences (*p* < 0.05). * Significant differences in means were found between men and women (Student’s *t*-test; *p* < 0.05). ^†^ Significant differences in means were found across all age groups (ANOVA; *p* < 0.05).

**Table 4:** Body weight, height and BMI of women aged 23 to 64 years.

Variables	*n*	Mean	SD	CV	p5	p10	p15	p25	p50	p75	p85	p90	p95
Body weight (kg) *^†^													
23–24	1993	55.53 ^a^	9.48	0.17	43.0	45.0	47.0	49.0	54.0	60.0	65.0	68.0	74.0
25–34	7554	56.46 ^b^	9.09	0.16	45.0	47.0	48.0	50.0	55.0	61.0	65.0	69.0	74.0
35–44	8915	57.75 ^c,d,e^	8.78	0.15	46.0	48.0	49.0	51.5	56.0	62.4	67.0	70.0	75.0
45–54	7551	58.06 ^c,d,e^	8.53	0.15	46.0	48.0	50.0	52.0	57.0	63.0	67.0	69.1	74.0
55–64	6888	57.86 ^c,d,e^	8.49	0.15	46.0	48.0	50.0	52.0	57.0	63.0	66.0	69.0	73.0
Total	32,901	57.42	8.81	0.15	45.0	47.4	49.0	51.0	56.0	62.0	66.0	69.0	74.0
Height (cm) *^†^													
23–24	1993	160.32 ^a,b^	5.81	0.04	151.0	153.0	154.6	156.0	160.0	164.0	166.0	168.0	170.0
25–34	7554	160.18 ^a,b^	5.68	0.04	151.0	153.0	154.0	156.0	160.0	164.0	166.0	167.5	170.0
35–44	8915	159.16 ^c^	5.46	0.03	150.0	152.0	154.0	155.5	159.0	163.0	165.0	166.0	168.0
45–54	7551	157.66 ^d^	5.40	0.03	149.0	151.0	152.0	154.0	158.0	161.0	163.0	164.8	166.9
55–64	6888	155.47 ^e^	5.41	0.03	147.0	149.0	150.0	152.0	155.0	159.0	161.0	162.0	164.1
Total	32,901	158.35	5.77	0.04	149.0	151.0	152.0	154.0	158.0	162.0	164.0	166.0	168.0
BMI (kg/m^2^) *^†^													
23–24	1993	21.59 ^a^	3.42	0.16	17.42	17.97	18.43	19.22	20.96	23.19	25.08	25.97	28.48
25–34	7554	22.01 ^b^	3.39	0.15	17.72	18.43	18.90	19.71	21.36	23.53	25.24	26.64	28.76
35–44	8915	22.80 ^c^	3.27	0.14	18.51	19.19	19.68	20.50	22.20	24.52	26.13	27.27	29.32
45–54	7551	23.36 ^d^	3.25	0.14	18.83	19.65	20.17	21.05	22.94	25.15	26.62	27.70	29.37
55–64	6888	23.94 ^e^	3.29	0.14	19.20	20.16	20.70	21.64	23.55	25.89	27.21	28.25	29.90
Total	32,901	22.91	3.39	0.15	18.29	19.05	19.63	20.54	22.38	24.80	26.37	27.48	29.36

BMI, body mass index; CV, coefficient of variation; SD, standard deviation; p with the ordinal number, percentiles. ^a,b,c,d,e^ Superscripts on the mean values represent Tukey’s test results. Means with the same letter represent that the mean values of the age groups have no significant difference between/among each other. In contrast, different superscript letters show significant differences (*p* < 0.05). * Significant differences in means were found between men and women (Student’s *t*-test; *p* < 0.05). ^†^ Significant differences in means were found across all age groups (ANOVA; *p* < 0.05).

**Table 5:** WC, HC and WHR of women aged 23 to 64 years.

Variables	*n*	Mean	SD	CV	p5	p10	p15	p25	p50	p75	p85	p90	p95
WC (cm) *^†^													
23–24	1993	71.80 ^a^	8.45	0.12	61.0	62.0	64.0	66.0	70.0	76.5	80.0	83.5	88.0
25–34	7554	73.23 ^b^	8.57	0.12	62.0	63.5	65.0	67.0	72.0	78.0	82.0	85.0	89.5
35–44	8915	75.27 ^c^	8.49	0.11	63.5	65.5	67.0	69.0	74.0	80.0	84.0	87.0	91.0
45–54	7551	77.01 ^d^	8.47	0.11	64.5	67.0	68.5	71.0	76.0	82.0	86.0	88.0	92.0
55–64	6888	79.41 ^e^	8.75	0.11	66.0	69.0	70.5	73.0	79.0	85.0	88.5	91.0	95.0
Total	32,901	75.86	8.88	0.12	63.0	65.0	67.0	69.5	75.0	81.0	85.0	88.0	92.0
HC (cm) *^†^													
23–24	1993	92.89 ^a^	7.01	0.08	82.5	85.0	86.0	88.0	92.0	97.0	100.0	102.0	106.0
25–34	7554	93.66 ^b^	6.87	0.07	84.0	86.0	87.0	89.0	93.0	98.0	100.5	103.0	106.0
35–44	8915	94.29 ^c,d^	6.50	0.07	85.0	87.0	88.0	90.0	94.0	98.0	101.0	103.0	106.0
45–54	7551	94.40 ^c,d^	6.40	0.07	85.0	87.0	88.0	90.0	94.0	98.0	101.0	103.0	106.0
55–64	6888	94.71 ^e^	6.42	0.07	85.0	87.0	88.0	90.0	94.0	99.0	101.0	103.0	106.0
Total	32,901	94.17	6.59	0.07	84.0	86.0	88.0	90.0	94.0	98.0	101.0	103.0	106.0
WHR *^†^													
23–24	1993	0.77 ^a^	0.06	0.08	0.69	0.70	0.71	0.73	0.76	0.81	0.84	0.85	0.89
25–34	7554	0.78 ^b^	0.06	0.08	0.69	0.71	0.72	0.74	0.77	0.82	0.85	0.86	0.89
35–44	8915	0.80 ^c^	0.06	0.08	0.71	0.73	0.74	0.76	0.79	0.84	0.86	0.88	0.90
45–54	7551	0.82 ^d^	0.06	0.07	0.72	0.74	0.75	0.77	0.81	0.85	0.88	0.89	0.92
55–64	6888	0.84 ^e^	0.06	0.07	0.74	0.76	0.77	0.80	0.83	0.88	0.90	0.92	0.95
Total	32,901	0.80	0.07	0.09	0.71	0.72	0.74	0.76	0.80	0.85	0.87	0.89	0.92

BMI, body mass index; CV, coefficient of variation; SD, standard deviation; p with the ordinal number, percentiles. ^a,b,c,d,e^ Superscripts on the mean values represent Tukey’s test results. Means with the same letter represent that the mean values of the age groups have no significant difference between/among each other. In contrast, different superscript letters show significant differences (*p* < 0.05). * Significant differences in means were found between men and women (Student’s *t*-test; *p* < 0.05). ^†^ Significant differences in means were found across all age groups (ANOVA; *p* < 0.05).

**Table 6:** BMI in different categories of men and women aged 23 to 64 years.

	Age Groups (Years)											
Variables	23–24		25–34		35–44		45–54		55–64		Total	
**Men** *	*n*	%	*n*	%	*n*	%	*n*	%	*n*	%	*n*	%
Underweight <18.5 (kg/m^2^)	136	5.73	184	2.20	82	1.00	66	1.06	51	1.12	519	1.75
Normal 18.5–23.9 (kg/m^2^)	1335	56.28	3886	46.47	2977	36.28	2194	35.40	1686	37.08	12,078	40.69
Overweight 24.0–26.9 (kg/m^2^)	523	22.05	2510	30.01	2963	36.11	2386	38.50	1759	38.69	10,141	34.16
Obese ≥27 (kg/m^2^)	378	15.94	1783	21.32	2183	26.61	1552	25.04	1051	23.11	6947	23.40
Total	2372		8363		8205		6198		4547		29,685	
**Women** *												
Underweight <18.5 (kg/m^2^)	315	15.81	812	10.75	443	4.97	258	3.42	183	2.66	2011	6.11
Normal 18.5–23.9 (kg/m^2^)	1297	65.07	5079	67.23	5827	65.36	4512	59.75	3591	52.13	20,306	61.72
Overweight 24.0–26.9 (kg/m^2^)	224	11.24	996	13.19	1650	18.51	1815	24.04	1981	28.76	6666	20.26
Obese ≥27 (kg/m^2^)	157	7.88	667	8.83	995	11.16	966	12.79	1133	16.45	3918	11.91
Total	1993		7554		8915		7551		6888		32,901	
**Pooled** *												
Underweight <18.5 (kg/m^2^)	451	10.33	996	6.26	525	3.07	324	2.36	234	2.05	2530	4.04
Normal 18.5–23.9 (kg/m^2^)	2632	60.30	8965	56.32	8804	51.42	6706	48.78	5277	46.14	32,384	51.75
Overweight 24.0–26.9 (kg/m^2^)	747	17.11	3506	22.03	4613	26.95	4201	30.55	3740	32.71	16,807	26.85
Obese ≥27 (kg/m^2^)	535	12.26	2450	15.39	3178	18.56	2518	18.31	2184	19.10	10,865	17.36
Total	4365		15,917		17,120		13,749		11,435		62,586	

BMI, body mass index. * Significant differences in means were found across all age groups (*x*^2^ test; *p* < 0.05).

**Table 7:** WC and WHR in different categories of men and women aged 23 to 64 years.

Age Groups												
Variables	23–24		25–34		35–44		45–54		55–64		Total	
Men *	*n*	%	*n*	%	*n*	%	*n*	%	*n*	%	*N*	%
WC < 90 (cm)	2015	84.95	6484	77.53	5654	68.91	4243	68.46	2955	64.99	21,351	71.93
WC ≧ 90 (cm)	357	15.05	1879	22.47	2551	31.09	1955	31.54	1592	35.01	8334	28.07
Total	2372		8363		8205		6198		4547		29,685	
**Women** *												
WC < 80 (cm)	1669	83.74	5979	79.15	6512	73.05	4976	65.90	3681	53.44	22,817	69.35
WC ≧ 80 (cm)	324	16.26	1575	20.85	2403	26.95	2575	34.10	3207	46.56	10,084	30.65
Total	1993		7554		8915		7551		6888		32,901	
**Pooled** *												
WC below cut-off	3684	84.40	12,463	78.30	12,166	71.06	9219	67.05	6636	58.03	44,168	70.57
WC above cut-off	681	15.60	3454	21.70	4954	28.94	4530	32.95	4799	41.97	18,418	29.43
Total	4365		15,917		17,120		13,749		11,435		62,586	
**Men** *												
WHR ≦ 0.90	2128	89.71	6942	83.01	5656	68.93	3639	58.71	2248	49.44	20,613	69.44
WHR > 0.90	244	10.29	1421	16.99	2549	31.07	2559	41.29	2299	50.56	9072	30.56
Total	2372		8363		8205		6198		4547		29,685	
**Women** *												
WHR ≦ 0.85	1774	89.01	6525	86.38	7231	81.11	5629	74.55	4143	60.15	25,302	76.90
WHR > 0.85	219	10.99	1029	13.62	1684	18.89	1922	25.45	2745	39.85	7599	23.10
Total	1993		7554		8915		7551		6888		32,901	
**Pooled** *												
WHR below cut-off	3902	89.39	13,467	84.61	12,887	75.27	9268	67.41	6391	55.89	45,915	73.36
WHR above cut-off	463	10.61	2450	15.39	4233	24.73	4481	32.59	5044	44.11	16,671	26.64
Total	4365		15,917		17,120		13,749		11,435		62,586	

WC, waist circumference; WHR, waist–hip ratio. * Significant differences in means were found across all age groups (*x*^2^ test; *p* < 0.05).

## Data Availability

The data used in this study is domestically available for public researches. Users may use the data at the appointed institute after the approval from the Sports Cloud: Information and Application Research Center of Sports for All, Sport Administration, Ministry of Education, Taiwan.
